# Comment on “Stroke awareness in a Brazilian Northeastern capital city and the burden of the COVID-19 pandemic”

**DOI:** 10.1055/s-0045-1802310

**Published:** 2025-02-04

**Authors:** Diogo Gonçalves dos Santos Martins, Thiago Gonçalves dos Santos Martins

**Affiliations:** 1 Universidade Federal de São Paulo, Escola Paulista de Medicina, Departamento de Oftalmologia, São Paulo SP, Brazil.; 2 Universidade Federal do Rio de Janeiro, Faculdade de Medicina, Departamento de Oftalmologia, Macaé RJ, Brazil.

Dear Editor,

In response to the article titled ‘Stroke awareness in a Brazilian Northeastern capital city and the burden of the COVID-19 pandemic,’ 1 published in your esteemed journal, which I consider a well-crafted and thoughtfully written paper, I would like to raise a few important points. The article examined general knowledge about stroke in a capital city of Northeastern Brazil and suggested new public policies. It emphasized the need for campaigns focused on educating the public about stroke risk factors and how to access emergency medical services in the state of Alagoas.

The use of artificial intelligence (AI) in the analysis of retinal imaging holds great potential as a public health screening policy for cerebrovascular accidents (CVA). Several studies have demonstrated that vascular changes detected in the retina can serve as risk biomarkers for cardiovascular diseases and strokes, with the retina acting as a ‘window’ into the vascular system.

A study 2 that analyzed 70 thousand retinal images found a correlation from 0.82 to 0.95 between vascular changes in the retina and other well-known cardiovascular risk factors, such as high blood pressure, elevated body mass index, and increased cholesterol levels. Another study 3 analyzed 65,144 participants and developed an algorithm capable of assessing a retinal vascular biomarker that predicts stroke, outperforming the Framingham risk scores for myocardial infarction and stroke. These findings suggest that retinal vascular alterations, such as tortuosity, can serve as non-invasive biomarkers to identify individuals at risk for cardiovascular diseases and stroke.

The implementation of AI can make retinal image analysis more efficient and accessible. In addition to enabling large-scale evaluations, these technologies have shown great promise in supporting the early diagnosis of cardiovascular diseases, particularly in regions with limited medical resources. By identifying vascular abnormalities with greater precision and speed than traditional methods, AI can facilitate the early detection of stroke risk, allowing for preventive interventions before clinical events occur.

As a public policy, population screening using AI for retinal image analysis can significantly reduce long-term treatment costs by lowering the incidence of strokes and their complications. Combined with other well-established cardiovascular risk factors, AI complements the diagnostic approach, providing a powerful tool to predict and prevent diseases through non-invasive analysis.

The use of AI in ophthalmology has shown tremendous potential in advancing our understanding of the pathophysiology of cardiovascular diseases by identifying new biomarkers. Moreover, this technology offers valuable support in diagnosing and monitoring these conditions, particularly in areas with limited healthcare resources. Due to its non-invasive nature, AI-based technologies are becoming increasingly important when integrated with more established exams for cardiovascular risk factors.

Therefore, incorporating AI into retinal imaging as a screening method has the potential to transform public health, offering an innovative and accessible solution for the prevention of strokes.
